# Bias-Variance Trade-Off in Continuous Test Norming

**DOI:** 10.1177/1073191120939155

**Published:** 2020-07-13

**Authors:** Lieke Voncken, Casper J. Albers, Marieke E. Timmerman

**Affiliations:** 1 University of Groningen, Groningen, Netherlands; 2 Tilburg University, Tilburg, Netherlands

**Keywords:** assumption violations, GAMLSS, model assumptions, model flexibility, skew Student *t* distribution, standard linear regression model

## Abstract

In continuous test norming, the test score distribution is estimated as a continuous function of predictor(s). A flexible approach for norm estimation is the use of generalized additive models for location, scale, and shape. It is unknown how sensitive their estimates are to model flexibility and sample size. Generally, a flexible model that fits at the population level has smaller bias than its restricted nonfitting version, yet it has larger sampling variability. We investigated how model flexibility relates to bias, variance, and total variability in estimates of normalized *z* scores under empirically relevant conditions, involving the skew Student *t* and normal distributions as population distributions. We considered both transversal and longitudinal assumption violations. We found that models with too strict distributional assumptions yield biased estimates, whereas too flexible models yield increased variance. The skew Student *t* distribution, unlike the Box–Cox Power Exponential distribution, appeared problematic to estimate for normally distributed data. Recommendations for empirical norming practice are provided.

Psychological tests are widely used to assess individuals in clinical and educational contexts. The test scores are often expressed as normed scores (Mellenbergh, 2011). Normed scores allow for an interpretation relative to the scores of the reference population, as defined for the test involved. For instance, the reference population of an intelligence test usually is the general population in the same country and of the same age as the testee involved. Normed scores are transformed versions of the raw scores, where the ordering within the reference population remains preserved. Popular examples include percentiles, normalized *z* scores and normalized IQ scores. The transformation rules to achieve normed scores can be directly obtained from the raw score distribution in the reference population. When norming a specific test, the population distribution is estimated based on scores from a normative sample that represents the reference population.

If the reference population depends on a continuous variable as age then, strictly speaking, there exists an infinite number of reference populations, within the age range of the test involved. Traditionally, the norms were derived for various successive age intervals (e.g., Wechsler Intelligence Scale for Children–III; Wechsler, 1991). In traditional norming, it is thus implicitly assumed that the test score distribution is the same across the whole age interval considered, and that this distribution changes as a step function of age. By noting that the performance on the intelligence test WAIS-R gradually changed with age rather than in a stepwise way, Zachary and Gorsuch (1985) considered the use of age intervals as suboptimal. Such gradual changes in test scores with age can be seen in developmental tests (e.g., van Baar et al., 2014), intelligence tests (e.g., Grob et al., 2018) and neuropsychological tests (e.g., Rommelse et al., 2018). Decreasing the width of the interval does not solve the issue, since the test score distribution estimates are then based on a smaller sample size per interval (Van Breukelen & Vlaeyen, 2005). Both issues with traditional norming are circumvented using the continuous norming approach.

## Continuous Norming

Continuous norming (e.g., Lenhard et al., 2018; Oosterhuis, 2017; Zachary & Gorsuch, 1985), unlike traditional norming, explicitly builds on the assumption that test score distributions change smoothly with age. It is thus more appropriate than traditional norming for those psychological tests that show this gradual change in test performance with age. The assumption of smoothly changing test score distributions is incorporated using regression modeling, in which the raw test score distribution is modeled as a function of predictor(s), as age. Because all observations within the normative sample—rather than subgroups—are used, continuous norming is more efficient than traditional norming (Oosterhuis et al., 2017). The approach to model a distribution as a function of a predictor is well-known from a standard linear regression analysis, assuming a normal distribution conditional on the predictor. For those conditional distributions that are nonnormal and/or heteroscedastic, more general regression models can be used to estimate normed scores. This can be done in nonparametric (Lenhard et al., 2018) and parametric ways.

A parametric regression framework that appeared useful in continuous norming of psychological tests (e.g., Grob et al., 2018; Tellegen & Laros, 2017; van Baar et al., 2014) are the generalized additive models for location, scale, and shape (GAMLSS; Rigby & Stasinopoulos, 2005). GAMLSS are regression models that include many different distribution types (Rigby et al., 2017). Their key feature is that the parameters that define the conditional distribution, can be modelled as a function of age. The four possible GAMLSS distribution parameters pertain to the location, scale, skewness, and kurtosis, while each specific distribution type is associated with a specific set of parameters. Both the distribution type, and the type(s) of relationships between the distributional parameters and age need to be selected before analysis. The distribution type fully determines the possible forms of the modeled distribution per age, and thus the nature of the transversal modeling. The type(s) of relationships fully determine the possible changes across age, and thus the nature of the longitudinal modeling. Because both aspects can be captured in many different ways, many different models can be created, varying from restricted models—with many and strict model assumptions—to flexible models—with few and loose assumptions.

The availability of many different models offers flexibility, yet makes model selection difficult. With flexibility we mean the possible range of data characteristics that can be captured by the estimated model. In the current context, the flexibility relates to both the transversal model (i.e., the distribution type) and the longitudinal model (i.e., the type(s) of relationships between age and the distributional parameters). In empirical norming with GAMLSS, one selects a candidate distribution by matching the nature of the raw test score distribution (i.e., considering the score range, their discrete or continuous character, and possible floor and ceiling effects), and candidate relationship(s) (e.g., considering their presumed smooth relationship). For a detailed strategy to regression-based norming with GAMLSS, we refer to Timmerman et al. (2019).

A central question in model selection is the amount of desired flexibility. Flexible models have the potential advantage of better fitting observed data than their restricted versions, because flexible models have a larger possible range of data characteristics that can be covered. However, to achieve the same precision of estimate (as reflected by e.g., the standard error [SE]), they require a larger sample size; further they are at the risk of overfitting (Hastie et al., 2009). In continuous test norming, it is unknown what the costs are of using a too restricted model versus the costs of using a too flexible model. A too restricted model means that the model contains too few parameters to adequately capture the population characteristics, and a too flexible model that it contains more parameters than strictly necessary.

## Standard Linear Regression Model

The standard linear regression model is a—rather restricted—variant of the GAMLSS models. Because this model forms the basis for more flexible models and is actually applied in continuous norming (e.g., Grober et al., 2015), we discuss its key features. The model is based on four assumptions: linearity, normality, homoscedasticity, and independence (e.g., Fahrmeir et al., 2013). The linearity assumption is that the model is linear in the parameters, implying a linear relationship between the predictor(s) in the model and the mean test score conditional on the predictor(s), like age. Possible nonlinear, yet smooth, relationships between predictor(s) and the mean test score can be accommodated by using transformed versions of the predictor(s) and/or test score. Examples are polynomials of the predictor(s), spline versions of the predictor(s) (e.g., Perperoglou et al., 2019), and a log transformation of the test score. The normality and homoscedasticity assumptions pertain to the normality of the conditional raw test score distributions (e.g., conditional on the predictor age), with a constant variance. The independence assumption is that the residuals (i.e., differences between the test scores and the conditional mean) are independent of one another. In contrast with the other three assumptions, violations of this assumption must be prevented with the sampling design followed in the normative study (i.e., individuals who make up the normative sample, should be sampled independently of each other). For this reason, we will only focus on the other three assumptions in this article.

In continuous norming practice, the relationship between the mean test score and the predictor is sometimes assumed to be linear (e.g., Agelink van Rentergem et al., 2018; Ganguli et al., 2010; Grober et al., 2015). Nonlinear, yet smooth relationships are modelled by including a second order polynomial of the predictor (e.g., Goretti et al., 2014; Kirsebom et al., 2019; Van der Elst et al., 2011) or high-order polynomials (e.g., Lenhard et al., 2018), or by using splines (e.g., Rommelse et al., 2018). Homoscedasticity and normality of the conditional score distribution are often assumed in norming practice (e.g., Goretti et al., 2014; Grober et al., 2015; Van Breukelen & Vlaeyen, 2005). Sometimes the tenability of these assumptions is assessed via the model residuals. Homoscedasticity seems to be mostly assessed with the Levene’s test (e.g., Llinàs-Reglà et al., 2013; Van der Elst et al., 2011), and normality with the Kolmogorov–Smirnov test (e.g., Goretti et al., 2014; Llinàs-Reglà et al., 2013; Van der Elst et al., 2011) or Q-Q plots (e.g., Kirsebom et al., 2019). Applying Levene’s test in the context of continuous norming is problematic as it can only be applied to test for homogeneity of variances of the score distributions within a certain group. Like in traditional norming, this requires discretization of the predictor variable(s). Van der Elst et al. (2011) applied the Levene’s test to four groups, which were formed by splitting up the predictor into four distinct segments (i.e., quartiles). In this way, the homogeneity of variances of the score distribution is assessed for only four predictor groups, and the variance is assumed to be equal within each group. Thus, homoscedasticity and normality checks in continuous norming are problematic in that these are assessed across pieces of or the full observed predictor space. If homoscedasticity and normality seem to hold for a given piece of the predictor space, one cannot rule out that the assumptions are violated locally. Furthermore, with such tests one cannot confirm that the assumptions are correct, but only fail to find evidence for violation of assumptions. On top of this, the consequences of violations of assumptions are often unclear and overestimated by applied researchers (Ernst & Albers, 2017; Williams et al., 2013).

## Assumption Violations in Continuous Norming Practice

We argue that continuous test norming practice often deals with nonlinearity, heteroscedasticity, and nonnormality. Bechger et al. (2009) already noted that the linearity assumption is probably unrealistic in practice. For intelligence and developmental tests (e.g., Grob et al., 2018; Kaufman & Kaufman, 2004; Wechsler, 2014) and neuropsychological tests (e.g., FEEST; Voncken et al., 2018), test scores that are based on the number of correct items typically increase strongly with age for young children, and this relationship diminishes or decreases as people get older (Ferrer & McArdle, 2004; McArdle et al., 2002). Also, we often see that the spread of the conditional score distribution varies with age (e.g., Grob et al., 2018; Tellegen & Laros, 2017). These aspects are to be covered in the longitudinal part of the norming regression model.

Floor and ceiling effects typically result in skewness of the conditional score distribution, possibly varying from positive skewness to negative skewness as a function of age. In addition, test scores based on response times typically result in a positively skewed conditional score distribution (Heathcote et al., 1991). These characteristics of psychological tests thus result in violations of the assumptions of homoscedasticity and normality. These aspects need to be covered in the transversal model, for example, by using a skew normal distribution.

**Figure 1.**
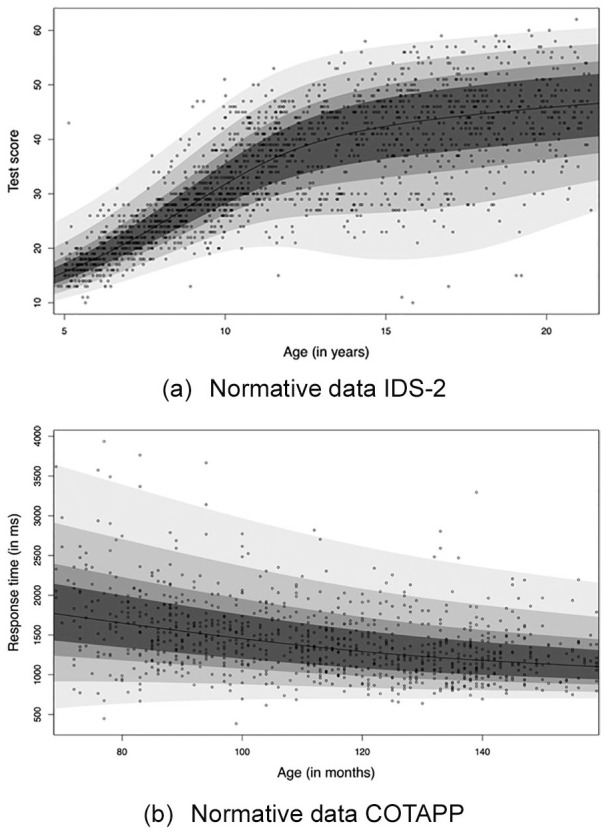
Estimated centile curves for the normative data of subtest “logical mathematical reasoning” of the Dutch IDS-2 (Grob et al., 2018) in panel (a), and subtest “Response time complex” of the COTAPP (Rommelse et al., 2018) in panel (b). *Note*. The observations are indicated with the black dots. The boundaries of the gray percentile bands represent percentiles 1, 5, 15, 25, 50 (black line), 75, 85, 95, and 99. IDS–2 = Intelligence and Development Scales–2.

Figure 1 illustrates nonlinearity, heteroscedasticity, and nonnormality in the observed test scores and their estimated continuous norming models of an intelligence test and a cognitive test. The observations and the percentile bands estimated as a function of age are shown for test scores of subtest “logical mathematical reasoning” of the Intelligence and Development Scales–2 (IDS-2; Grob et al., 2018) in panel (a), and for response times of subtest “Response time complex” of the Cognitive Test Application (COTAPP; Rommelse et al., 2018) in panel (b). To create the percentile curves for these subtests, the median, variation, skewness, and kurtosis of the Box–Cox Power Exponential (BCPE) distribution (Rigby & Stasinopoulos, 2004) were estimated as a smooth, possibly nonlinear function of age using P-splines. The BCPE distribution has distributional parameters µ, σ, ν, and τ, which relate to the median, variation, skewness, and kurtosis, respectively. The default link functions were used: the identity link function for µ and ν, and the log link function for σ and τ. Splines (for a review, see Perperoglou et al., 2019) are polynomial functions, which are used in regression to achieve a smooth estimated function. P-splines are a variant that is numerically stable, easy to implement and requires only a single penalty parameter to control the smoothness of the complete function (Eilers & Marx, 1996). Both models in Figure 1 showed good fit, as indicated by their associated worm plots (Van Buuren & Fredriks, 2001), which are detrended Q-Q plots, possibly conditional on ranges of the predictor(s).

Figure 1(a) shows a nonlinearly increasing relationship between the median intelligence test score and age, age-depending variance of the conditional score distribution, and age-depending skewness and kurtosis of the conditional score distribution (as reflected by the nonsymmetric width of the percentile bands). Figure 1(b) shows a nonlinearly decreasing relationship between the median response time and age, and age-depending variance, skewness and kurtosis of the score distribution. The associated continuous test norming models appeared to fit the normative data well, but it is unknown how much flexibility is optimal.

In general, using more flexible models allows for more accurate (i.e., with smaller bias) estimation of the percentiles, because the model can fit the observed data at least as well as a more constrained variant. However, flexibility comes with risks of overfitting and large sampling variability (Everitt, 1998; Sammut & Webb, 2010). This sampling variability can be reduced by increasing the sample size, but this is expensive and larger samples are not always available. If the decrease in bias by using a more flexible model is small relative to the increase in required sample size, the increased flexibility might not be worth it.

Oosterhuis (2017) investigated consequences of violating the linearity, homoscedasticity, and independence assumptions in continuous test norming. In a simulation study only considering the standard linear regression model, Oosterhuis found that violations of these assumptions introduced bias in the percentile estimates, but they did not affect the precision of the percentile estimates. In this article, we extended the study by Oosterhuis and investigated in a simulation study (1) the costs in terms of the bias of using restricted models in the presence of violations of (combinations of) model assumptions and (2) the costs in terms of the sampling variability of using more flexible GAMLSS models than strictly needed to properly describe the population data. Thus, we investigated the balance between the bias and variance in normed scores related to the model flexibility. Based on this, we explored how robust the various models are and thus how sensitive the issue of model flexibility is.

## Simulation Study

The simulation study was performed in R (version 3.6.1; R Core Team, 2019). We used version 5.1-6 of the gamlss package (Rigby & Stasinopoulos, 2005). The R code can be found on the Open Science Framework via https://osf.io/hwme5/.

### Research Questions and Hypotheses

In this simulation study, we investigated how model flexibility relates to bias (i.e., accuracy), variance (i.e., precision), and total variability (as expressed by the root mean square error (RMSE)) in the estimates of normalized *z* scores based on regression modeling under empirically relevant conditions. More specifically, we investigated (1) what the consequences of transversal and/or longitudinal assumption violations in continuous test norming are for the bias in the normalized *z* score estimates, (2) how much precision is lost (i.e., how much the variance increases) when using a more flexible model using the same sample size, and (3) what the net effects on the total variability in estimates (i.e., RMSE) are of assumption violations and flexibility.

We selected the normalized *z* score as the statistic of interest, because it is often used in norming practice. Furthermore, it is not limiting, because other normed scores, as percentiles and normalized IQ scores, can be directly derived from normalized *z* scores. The bias concerns the center of the sampling distribution. Specifically, the normalized *z* score estimate of an observed test score at a certain age is unbiased if the mean of its sampling distribution equals the population normalized *z* score. The variance of the sampling distribution indicates the degree of variability in estimated normalized *z* scores of an observed score at a certain age across samples. A larger sample size is associated with a smaller variance (Moore et al., 2012). A desirable property of a fitting model (i.e., the estimated model complies with the population model or is less constrained) is that it is unbiased, or, at least consistent (Lindgren, 1993).

In the study, we investigated to what extent the bias and variance of the normalized *z* score estimates are influenced by three factors. Factor (1) is the kind of population model, expressed via their transversal and longitudinal nature. Factor (2) is the sample size *N*. Factor (3) is the flexibility (i.e., too flexible, true, and too strict) of the transversal and longitudinal nature of the estimation model, in relation to the transversal and longitudinal nature of the population model.

In line with the findings of Oosterhuis (2017), we expected that using a too strict model would result in higher bias compared with the true and too flexible models, but would keep the precision of the normalized *z* score estimates at the same or a lower level. Furthermore, we expected that larger differences between the population model and the stricter estimated models would result in a higher bias. With respect to the variance, we expected the variance to decrease as the sample size increases, and the variance of a too flexible model to be higher than of the true model. We did not have hypotheses on the effects on deviations in terms of the transversal and longitudinal nature. With respect to the RMSE, we expected the RMSE of the true model to be lower than of the too strict and too flexible models. We expected so because we designed the study such that the true model differed substantially from the too strict and too flexible models. This implied that we expected the bias introduced with using the too strict model would not be compensated with the decrease in variance. Furthermore, we expected the too flexible models to have similar or somewhat higher levels of bias than the true model, and with an increased variance.

### Design

As the starting point for our population models (Factor [1]), we used a model estimated based on the normative data of subtest “logical mathematical reasoning” of the Dutch IDS-2 (Grob et al., 2018). The test scores show non normality, heteroscedasticity, and smooth, nonlinear effects of age. This pattern is more often found in intelligence and developmental tests, in that test scores typically increase rapidly with age for young children, and this relationship decelerates with age as they get older (e.g., Ferrer & McArdle, 2004; Grob et al., 2018; Kaufman & Kaufman, 2004; McArdle et al., 2002).

As the transversal model for the normative data, we selected the skew Student *t* distribution (Fernandez & Steel, 1998), as reparametrized by Würtz et al. (2006). This distribution has four distributional parameters, namely µ for the mean, σ for the standard deviation, and ν and τ, which are related to the skewness and kurtosis, respectively. We used the default link functions (i.e., identity link for µ, log link for σ and ν, and ln[τ *−* 2] for τ). We selected the skew Student *t* distribution, because we expected that it would capture the nonnormal and heteroscedastic normative data reasonably well, and because based on this model we could easily make normal and/or homoscedastic model variants. This is so because the skew Student *t* distribution simplifies to the normal distribution when ν = 1 and τ = ∞, and its σ parameter equals the standard deviation (this is unlike, e.g., the BCPE distribution). As the longitudinal model for the normative data, we used orthogonal polynomials of age.

**Figure 2.**
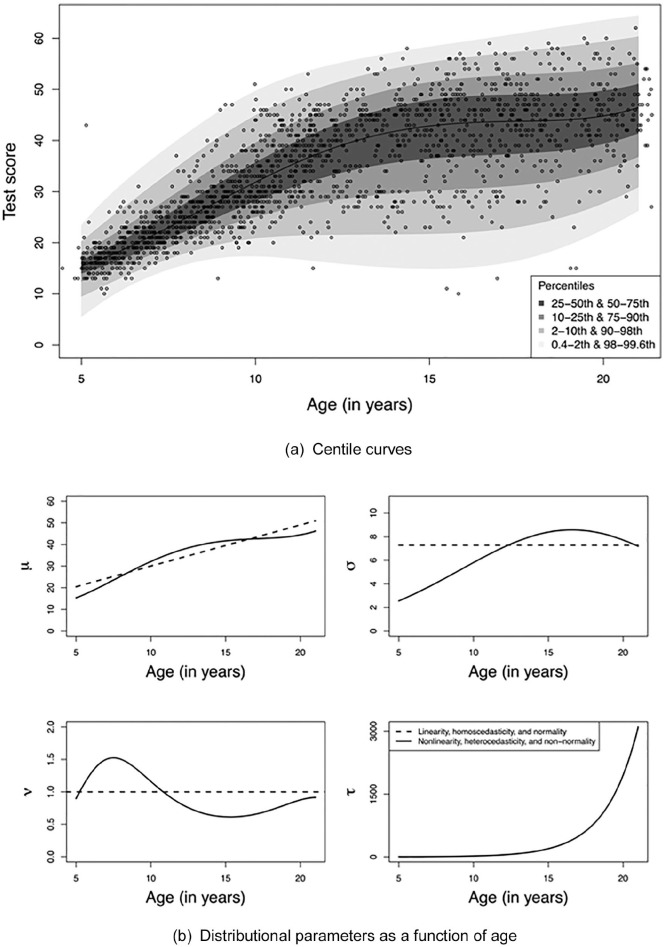
Centile curves (panel a) and distributional parameters of the skew Student *t* distribution as polynomial function of age (solid lines; panel b) for the IDS model. *Note*. The dashed lines in panel (b) indicate the conditions with linearity in µ, homoscedasticity (i.e., σ is constant, and normality (i.e., ν = 1; τ = ∞ is not depicted). IDS–2 = Intelligence and Development Scales–2.

To select the model for the normative data, the IDS model for short, we fitted a series of presumably fitting models, selected the model with the lowest Bayesian information criterion (BIC; Schwarz, 1978), and checked model fit using the worm plot. Specifically, we fitted the skew Student *t* distribution, using all possible combinations of orthogonal polynomials of age, with polynomial degrees ranging from 0 up to 5 for distributional parameters µ, σ, and ν, and polynomials degrees from 0 up to 2 for distributional parameter τ, thus considering 63 * 3 = 648 models in total. The selected IDS model includes polynomials of age up to degree 4 for both µ and ln ν, and up to degree 2 for ln σ, and degree 1 for ln (τ − 2). Figure 2 depicts the centile curves (panel a) and the distributional parameters as a function of age (panel b) for the IDS model. Distributional parameter µ corresponds to the mean value of the conditional test score distribution, σ corresponds to the standard deviation of the conditional score distribution, and ν and τ correspond to the skewness and kurtosis. The closer ν is to 1 and the higher τ is, the closer the conditional score distribution resembles a normal distribution.

The IDS model involves in the longitudinal model nonlinearity for µ, and in the transversal model heteroscedasticity and nonnormality, which are depicted with solid lines in panel (b) of Figure 2. Based on this, we defined a linear variant for µ, a homoscedastic variant (i.e., σ constant), and a normal variant (i.e., ν = 1; τ = ∞), as depicted with dashed lines in panel (b) of Figure 2. By using all possible combinations of (non)linearity, homoscedasticity/heteroscedasticity, and (non)normality, we defined 2^3^ = 8 population models.

Normative samples were randomly generated from each population model, with sample size *n* = 500, 1,000, and 2,000 (Factor 2)—which are in the typical range of what is being used in practice. The age values were fixed to *N* evenly spaced values in the range [5, 21]. For Factors (1) and (2), we used a fully crossed design, involving *R* = 1,000 replications. This resulted in 8 (population model) × 3 (*N*) *×* 1,000 (*R*) = 24,000 generated normative samples.

Factor (3) is the flexibility (i.e., too flexible, true, and too strict) of the transversal and longitudinal nature of the estimation model, in relationship to the transversal and longitudinal nature of the population model. Specifically, for each sample, we estimated the true model, and as far as applicable, the transversal and/or longitudinal strict models, and the transversal and/or longitudinal flexible models.

As the true model, we used the skew Student *t* distribution for the transversal model (i.e., population and estimation models equal), and the longitudinal model with a linear effect for conditions with a linear population model, and P-splines for the conditions with a nonlinear population model (Eilers & Marx, 1996; i.e., population and estimation model different, but expected to yield similar results); therefore we denote this model as True[linear/splines] in what follows. To keep the simulation study feasible, we used a fixed number of 6 knots, rather than to each simulated data set tailored number. We selected the number of knots such that it was optimal (i.e., lowest Akaike information criterion and lowest BIC) for the most complex population model (i.e., nonlinearity, heteroscedasticity, and nonnormality). The smoothing parameter of the P-splines was automatically selected using the generalized Akaike information criterion (GAIC) method with penalty 5, which was optimal (i.e., lowest BIC) for the most complex population model given the selected 6 knots. To assess to what extent the P-splines indeed yield similar results as polynomials would achieve, we estimated for the most flexible nonlinear population model (i.e., the IDS-2 population model) also a model involving polynomials; this model will be denoted as True[poly].

The strict transversal model variant involved homoscedasticity and normality, and the strict longitudinal model variant involved a linear model for µ. The flexible transversal model variant involved heteroscedasticity and nonnormality, and the flexible longitudinal model variant involved splines for µ.

To examine to what extent the simulated data could be approximated with a different distribution than the population one (i.e., skew Student *t* distribution), we also estimated a BCPE distribution using default link functions, and P-splines. To accommodate for possible negative simulated test scores, the BCPE model was estimated on the test scores plus 100 for each value, and the estimated values were backtransformed accordingly.

We estimated all applicable combinations of models. For example, the IDS-2 population model involved nonlinearity, heteroscedasticity and nonnormality. Because this is the most flexible variant that we consider, only the strict estimation model variants are applicable next to the true model.

### Outcome Measures

The main outcome measures were the bias, variance, and the RMSE of the normalized *z* score estimate, at age value *i* and test score *j*. RMSE_*ij*_ is a combination of bias and variance (i.e., $RMSE_{ij}=\sqrt{variance_{ij}+bias_{ij}^{2}}$ ). It expresses how much the estimated normalized *z* scores deviate from the population normalized *z* score, due to sampling variability (i.e., variance) and a systematic difference (i.e., bias). If an increase in model flexibility resulted in an increased RMSE_*ij*_, this would indicate that the increase in variance is larger than the decrease in squared bias for that age value and test score.

We evaluated the three outcome measures per age value and test score across the *R* = 1,000 replications, for *I* (=1,000) equally spaced age values across the full age range [5, 21], and *J* (= 100) test scores corresponding to (conditional) population *z* scores in the range [−3, +3]. Conditional test scores outside this range (i.e., deviating more than 3 *SD*s from the mean score) are sometimes not reported in practice (e.g., in the IDS-2 intelligence test; Grob et al., 2018) because the uncertainty in those scores is considered to be too large and therefore not relevant in our outcome measures. The normalized *z* score estimates were derived from the percentiles of the estimated test score distribution conditional on age via the inverse normal distribution. Small differences in extreme estimated percentiles resulted in large differences in extreme *z* scores, and very extreme estimated percentiles resulted in *|z*ˆ*|* = ∞. This is why we bounded the estimated normalized *z* scores to the range [−5, +5], which is very extreme in practice.

Thus, the bias, variance, and RMSE of *z_ij_*, the normalized *z* score at age value *i* and test score *j*, were computed as

$$bias_{ij}=\frac{1}{R}\sum_{r=1}^{R}({\hat{z}}_{ijr}-z_{ij})={\overset{-}{z}}_{ij}-z_{ij},$$

$$variance_{ij}=\frac{1}{R}\sum_{r=1}^{R}{\left({\hat{z}}_{ijr}-{\overset{-}{z}}_{ij}\right)}^{2},and$$

$$RMSE_{ij}=\sqrt{\frac{1}{R}\sum_{r=1}^{R}{\left({\hat{z}}_{ijr}-z_{ij}\right)}^{2}}.$$

In addition to our main outcome measures (i.e., bias, variance, and RMSE), we evaluated the convergence of all estimated models. We defined two types of nonconverging models: (1) models that could not be estimated and (2) models that were estimated with the maximum number of iterations, which we fixed to 2,000 iterations. The computations of the main outcome measures were based on all the models that could be estimated, thus including those that were estimated with the maximum number of iterations.

## Results

**Figure 3.**
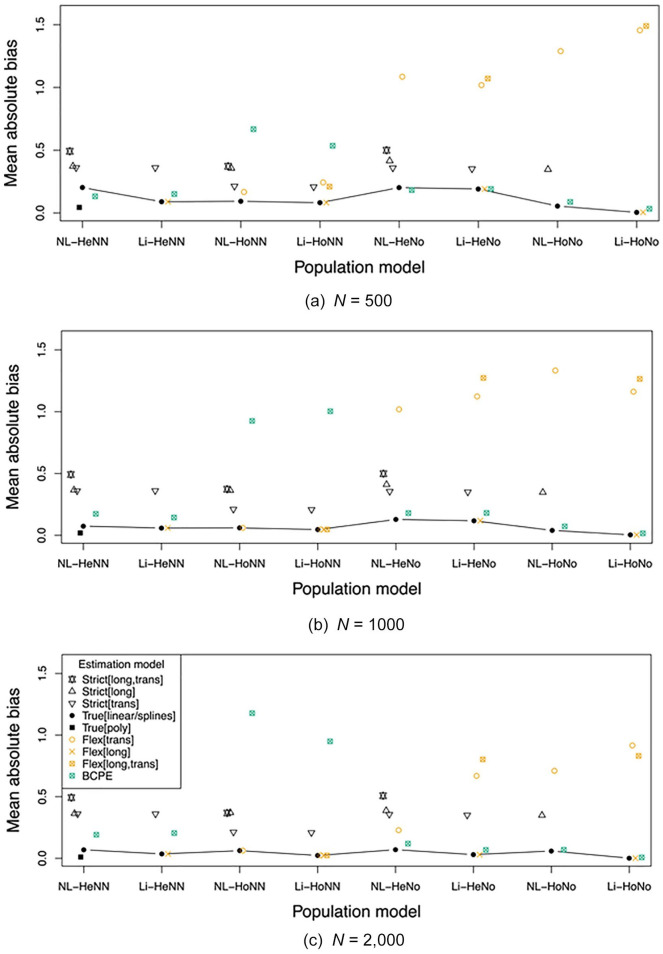
Mean absolute bias across all ages and test scores evaluated, per population model and estimation model, for *n* = 500 (a), *n* = 1,000 (b) and *n* = 2,000 (c). *Note*. Estimation model is too strict (Strict), or too flexible (Flex), related to the trans(versal) and/or long(itudinal) model; True[linear] and True[poly] equal, and True[splines] approximates the population model. NL = nonlinear; Li = linear; Ho = homoscedastic; He = heteroscedastic; No = normal; NN = nonnormal; BCPE = Box–Cox Power Exponential model.

**Figure 4.**
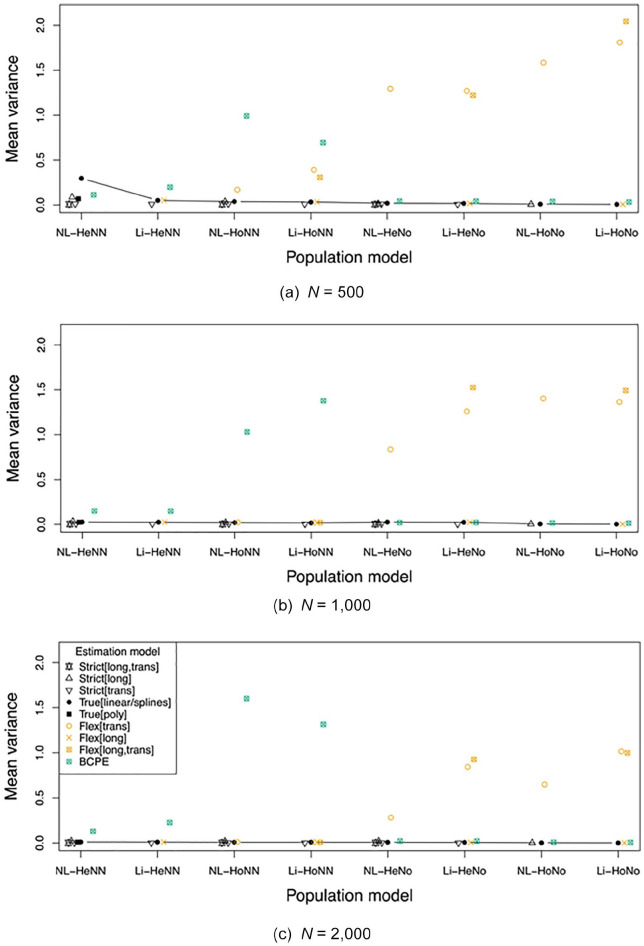
Mean variance across all ages and test scores evaluated, per population model and estimation model, for *n* = 500 (a), *n* = 1,000 (b) and *n* = 2,000 (c). *Note*. Estimation model is too strict (Strict), or too flexible (Flex), related to the trans(versal) and/or long(itudinal) model; True[linear] and True[poly] equal, and True[splines] approximates the population model. NL = nonlinear; Li = linear; Ho = homoscedastic; He = heteroscedastic; No = normal; NN = nonnormal; BCPE = Box–Cox Power Exponential model.

**Figure 5.**
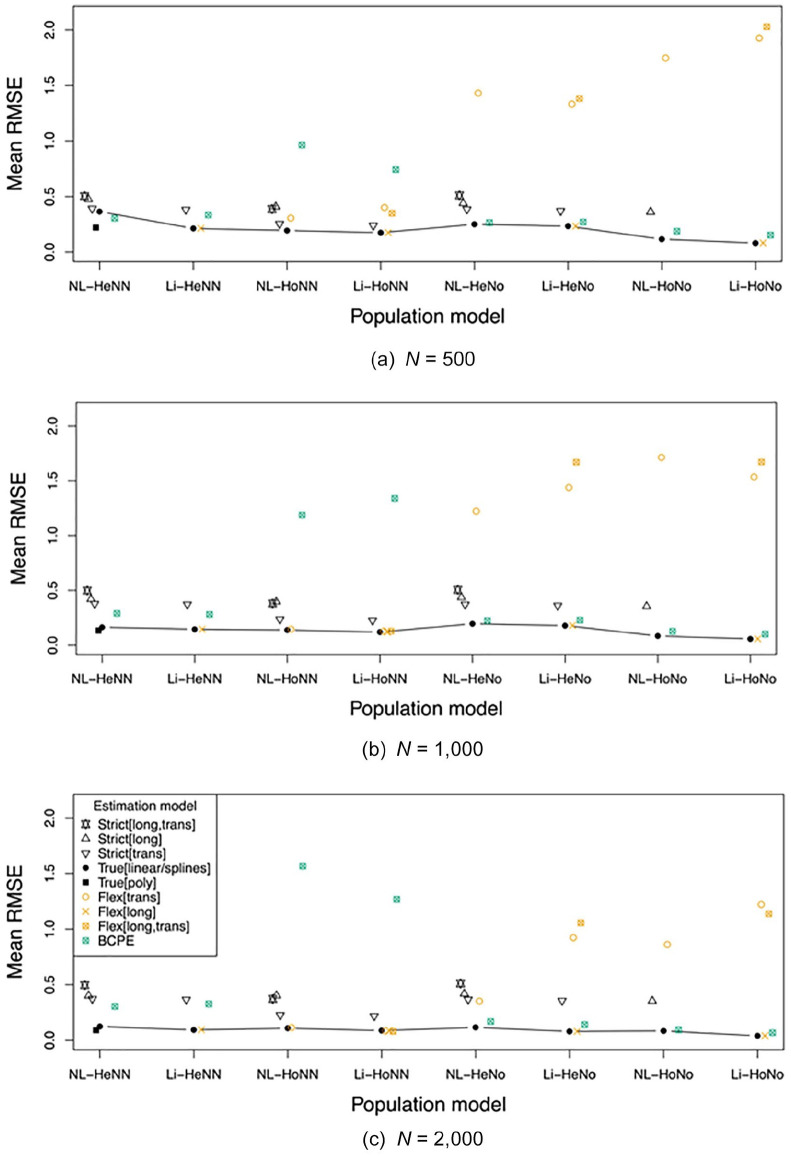
Mean RMSE across all ages and test scores evaluated, per population model and estimation model, for *n* = 500 (a), *n* = 1,000 (b) and *n* = 2,000 (c). *Note*. Estimation model is too strict (Strict), or too flexible (Flex), related to the trans(versal) and/or long(itudinal) model; True[linear] and True[poly] equal, and True[splines] approximates the population model. RMSE = root mean square error; NL = nonlinear; Li = linear; Ho = homoscedastic; He = heteroscedastic; No = normal; NN = nonnormal; BCPE = Box–Cox Power Exponential model.

The key results are presented in Figures 3 to 5. These show the absolute bias, variance, and RMSE of the normalized *z* scores, as averaged across all ages and test scores evaluated, per combination of population model and estimation model. The associated numbers with their *SE*s are provided in the Supplementary Material (see https://osf.io/hwme5/). The order of population models is the same in the three figures, with the most flexible nonlinear population model (i.e., the IDS-2 population model) first, and the most restricted population model (i.e., linear regression model with homoscedasticity) last. The *SE*s of the outcome measures are very small relative to the differences in the average outcome variables between conditions. Thus, we can reliably interpret the means represented. The Supplementary Figure S1, which is available via the same OSF link, illustrates for one condition (i.e., population model without assumption violations in combination with the “strict” estimation model, for *n* = 500, age 5, and *z* score = 0) that 1,000 replications were more than enough because convergence of the RMSE measure was already reached after about 700 replications.

To ease the reading of the results, we present them associated to the hypotheses stated in the section “Research questions and hypotheses.”

### Bias

We expected that using a too strict model would result in higher bias than using the true models and the too flexible models. Comparing in Figure 3 all Strict, True, Flex, and (too flexible) BCPE estimation models for all population models shows that this is indeed the case at all sample sizes for the conditions with (1) the normal (No) population models, estimated with the BCPE model; and (2) the nonnormal (NN) population models, estimated with the Flex models (i.e., involving the Skew Student *t* distribution), and for the condition involving heterogeneity, estimated with the BCPE model. Deviations from the expectation are found in (3) the normal (No) population models, estimated with the Flex models, which are too flexible in the transversal model (i.e., Flex[trans] and Flex[long,trans]); and (4) the nonnormal population models involving homoscedasticity (Ho), estimated with the BCPE model.

We further expected that a larger difference between the population model and the strict estimation model would result in a higher absolute bias. Comparing in Figure 3 the (most restricted) Strict[long,trans] model with the (less restricted) Strict[long] and Strict[trans] models for each population model and sample size show that this is indeed seen at all cases considered.

### Variance

We expected that using a too strict model would result in comparable or lower variance when compared with the true models and the too flexible models. Comparing in Figure 4 all Strict, True, Flex, and BCPE model variants shows that this is indeed the case, for all population models and sample sizes.

We further expected the variance to decrease as the sample size increases. Comparing in Figure 4 the three samples sizes per combination of population and estimation models learns that this is generally the case, with the following exception: (1) the nonnormal population models, estimated with the BCPE model, with substantial deviations in the homoscedastic (Ho) condition. Inspection of the means (to be found in the Supplementary Material [see https://osf.io/hwme5/]) learns that small deviations are also found in (2) the normal population models with heteroscedasticity and nonlinearity (NL-HeNo), estimated with the Strict[long] and True[linear/spline] models; and (3) the normal population models with heteroscedasticity and linearity (Li-HeNo), estimated with the True[linear/spline], Flex[long] and Flex[long, trans] estimation models.

We finally expected the variance of a too flexible model to be higher than of the true model. Comparing in Figure 4 the Flex and BCPE model variants to the True[linear/splines] model and the True[poly] model (if available) per population condition learns that this is indeed seen at all cases considered. Note that the larger variance of the True[linear/splines] model than the BCPE model in the NL-HeNN condition at sample size *n* = 500 does not indicate an exception, because the True[linear/splines] model still deviates from the population model, in that splines rather than polynomials are used. The True[poly] model equals the population model, and their associated variance is indeed lower than of the BCPE model.

### RMSE

We expected the RMSE of the true model to be lower than that of the too strict and too flexible models. Comparing in Figure 5, the True models with all other estimation models shows that this is indeed the case, for all population models and sample sizes. For the Flexible models it appeared that the RMSE of the Flexible models was comparable or a bit higher than the RMSE of the True models, except for two notable exceptions: (1) the BCPE model in the non normal homoscedastic population conditions (HoNN), and (2) the Flex estimation models in the Normal population conditions (No). For the Strict models it appeared that the RMSE of Strict models were consistently higher than of the True models, and also of the Flexible models, except for the two exceptions as described in the previous line.

**Figure 6.**
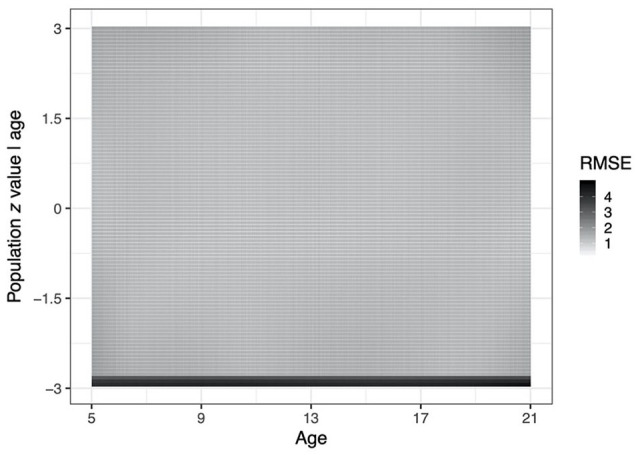
Heat plot of the RMSE (root mean square error) of the estimated *z* values over all replications for each combination of age and population *z* score conditional on age, for the most restricted population model, with linearity, homoscedasticity and normality (Li-HoNo), at a sample size of *n* = 500, and using the most flexible estimation model, i.e., the Flex[long,trans] model.

To offer some illustration on how much the RMSE depends on the region in the observed predictor space, Figure 6 shows a heat map for all combinations of age values and population *z* scores conditional on age, for the condition with the highest mean RMSE. This condition is the most restricted population model (i.e., the linear regression model with homoscedasticity and normality) combined with the most flexible estimation model (i.e., the skew Student *t* distribution, using P-splines), at the sample size of *n* = 500. The heat map shows that the RMSE has rather high values for the extremely low population *z* values (say below −2.7), and rather low values for all other *z* values, consistently across all ages. The P-splines thus yield a consistent fit across all ages, while the skew Student *t* distribution yield a rather poor estimation at the lower tail of the population distributions at all ages. Inspection of the estimated scores revealed that the low *z* scores are underestimated.

### Nonconvergence

Per condition (i.e., all 43 combinations of population model and estimation model, for 3 sample sizes), we assessed the frequency (out of 1,000 replications) of (1) models that could not be estimated, and (2) models that were estimated with the maximum number of 2,000 iterations. The full tables are available in the online Supplementary Material (https://osf.io/hwme5/). Across all 129 conditions, the maximal number of models that could not be estimated ranged from 0 (<0.01%) to 4 (0.04%), and the maximal number of models estimated with the maximal number of iterations ranged from 0 (<0.01%) to 60 (6.0%). Thus, nonconvergence poses little to no thread to the robustness of our results.

### How to Diagnose Underfitting and Overfitting in Empirical Practice Using Visual Diagnostics

In practice, the population model is unknown and one has to estimate the norming model based on a normative sample. As a result, underfitting and overfitting of the normative sample data are serious risks. We illustrate based on empirical normative data (*N* = 1,654) of the subtest “logical mathematical reasoning” of the Dutch IDS-2 (Grob et al., 2018) how centile curves and worm plots (Van Buuren & Fredriks, 2001) can be used to visually diagnose underfitting and overfitting. We estimated three models: a very strict model (i.e., assuming linearity, homoscedasticity, and normality), a properly fitting model (i.e., using the SST distribution with P-splines, with the smoothing parameter selected by the BIC), and a very flexible model (i.e., same model as the properly fitting model, yet selecting the smoothing parameter by the GAIC (0.1), which entails a much smaller penalty than the BIC does on the model complexity).

**Figure 7.**
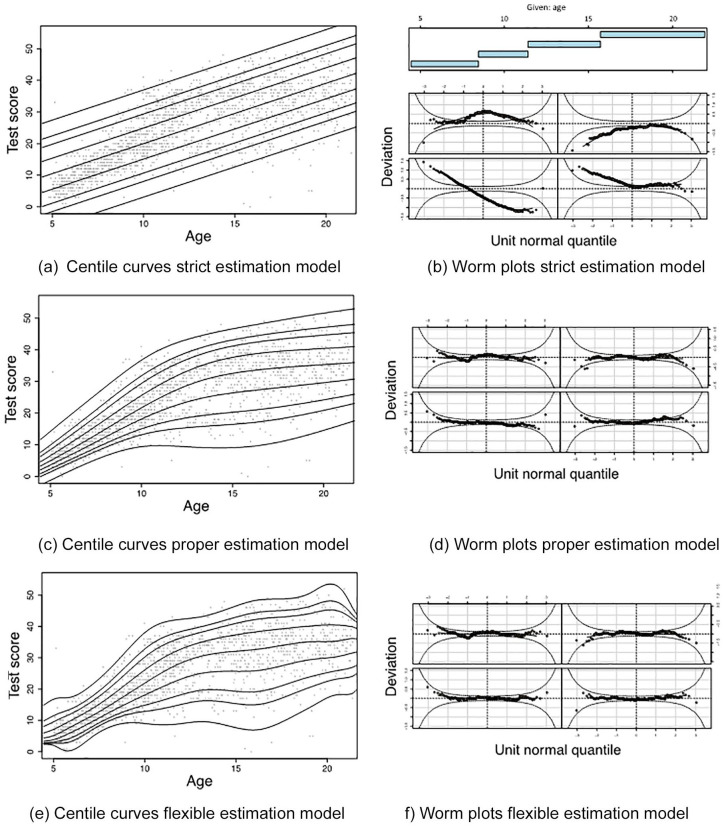
Centile curves (left panels) and worm plots (right panels) for a strict model (assuming linearity, homoscedasticity, and normality), a model with proper fit (SST[BIC]), and a flexible model (SST[GAIC (0.1)]) as estimated for empirical normative data of subtest “logical mathematical reasoning” of the Dutch IDS-2 (Grob et al., 2018). *Note*. The nine centile curves within each left panel correspond to percentiles 1, 5, 10, 25, 50, 75, 90, 95, 99 as a function of age, and the grey dots indicate the sample scores. BIC = Bayesian information criterion; GAIC = generalized Akaike information criterion.

Figure 7 shows the centile curves and the observed test scores as a function of age and the corresponding worm plots (Van Buuren & Fredriks, 2001) for the three estimation models. As can be seen in Figure 7, the centile curves of the strict estimation model in panel (a) all increase linearly with age. Note that the median increases linearly with age due to the linear nature of the longitudinal model only, and that all percentiles increase linearly with age due to both the linear nature of the longitudinal model and the age independent nature of the transversal model. This model clearly results in underfitting because the centile curves do not follow the general pattern in the normative data, which corresponds with the large deviations from zero (i.e., the horizontal dotted lines) in the worm plots in panel (b). The centile curves of the properly fitting and flexible estimation models, in panels (c) and (e) respectively, seem to follow the pattern in the normative data much better. This is shown by the small deviations from zero in the worm plots in panels (d) and (f). However, the centile curves of the flexible estimation model seem too wiggly, especially for the extreme percentiles, thereby most likely overfitting the sample data. Theoretically, the relationship between the percentiles and age is expected to be smooth, and not wiggly. This example illustrates that underfitting is best detected using the worm plots, and overfitting is best detected using the centile curves in combination with theoretical expectations about the relationship between the percentiles and the predictor(s).

## Discussion

The results of the simulation study largely supported all hypotheses stated. That is, in our simulation study it generally appeared that the use of a too strict estimation model results in higher bias compared with using the true or a too flexible models, while the precision of the normalized *z* score estimates is at the same or a lower level. Also, a larger difference between the population model and the too strict estimation model results in higher bias. Furthermore, the variance of the estimates of a too flexible model is higher than of the true model, and the variance decreases with increasing sample size. Finally, the RMSE of the true model is lower than of the too strict and too flexible models.

In our simulation, we found some notable results that deviated from our stated hypotheses. We carefully considered these results, and inspected estimated models of a sample of replicates. In our view, the exceptions found stem from two difference sources. First, the estimation with the skew Student *t* distribution appeared to be problematic in samples drawn from a normal population distribution. Theoretically, the skew Student *t* distribution with distributional parameter τ equal to ∞ equals a normal distribution. We presume that the estimation issues occurred in samples drawn from a normal distribution, because the estimated parameter τ may thus become very large, and with large fluctuations across samples.

Second, the estimation with the BCPE distribution appeared to be problematic, with the largest effects seen in samples drawn from nonnormal populations involving homoscedasticity. We presume that these estimation issues are due to the use of too flexible longitudinal models for the parameters σ, ν, and τ. We used P-splines with a fixed number of knots to keep the simulation study feasible, and this number was based on optimal knot selection for the most complex population model (i.e., with nonlinearity, heteroscedasticity, and nonnormality), thus requiring the largest degree of flexibility. For, for example, a sample from a homoscedastic population, this is obviously too flexible for the spread parameter σ, because an intercept only would be sufficient already.

### Practical Recommendations

Our simulation study has clear implications for empirical practice. In empirical norming with GAMLSS, one models the distribution of test scores, where the distributional parameters are modelled as functions of the predictor. The best choice would be to use a distribution with specific functions of the predictor that would fit the population distributions well. Thus, the estimation model applied may deviate from the exact population model, as long as the approximation is well. A more constrained model would result in biased estimates, while a more flexible model would result in a larger variance.

In practice, one obviously does not know which model fits well at the population level. The way to proceed is to select a candidate distribution and candidate relationship, based on, as far as possible, knowledge about the nature of the raw test scores and relationships between the predictor and the distributional parameters (e.g., smoothness). For scores that presumably can be fitted with a continuous distribution, the BCPE distribution seems to be a safer choice than the skew Student *t* distribution, given the estimation problems of the latter that we encountered for (about) normally distributed data. The specific model parameters (e.g., degrees of the polynomials, or penalty parameters of the P-splines) can then be selected by fitting a series of models (e.g., using all combinations of polynomial degrees in a certain range), and selecting the optimal one based on an information criterion (e.g., BIC; Schwarz, 1978). In this way, the model fits the sample data as closely as possible (i.e., to prevent underfitting), while penalizing the model complexity (i.e., to prevent overfitting). In a similar way, the smoothing parameter in the P-splines balances underfitting against overfitting. Other regularization/penalization methods to prevent overfitting in model selection are available, such as cross-validation (e.g., see Hastie et al., 2009).

Herewith, we advise to take a substantial number of maximal iterations (say, 2,500), and possibly increase that number if the maximum number of iterations would be needed, at least for the finally selected model. For the latter model, it is also important to visually inspect its model fit, using worm plots (Van Buuren & Fredriks, 2001), and plots of the centile curves and empirical observations (e.g., as in Figure 1), as illustrated in the Results section. This visual inspection should be guided by theoretical expectations about the relationship between the percentiles and the predictor(s). While underfitting and overfitting cannot be prevented completely in practice, this helps keeping their degree minimal.

In this article, we considered the normed scores associated with observed test scores. These test scores serve as the best estimates of the latent trait that is measured with the test, and thus are point estimates. In using test scores for individual decisions, it is important to acknowledge the uncertainty in the point estimate. This can be done by using a 95% confidence interval for the raw test score, which thus expresses the uncertainty due to test unreliability. The interval can be based on classical test theory, or measurement models as item response theory-based models. Once the boundaries of the confidence interval for raw test score are known, they can be converted to the boundaries of the normed scores, using the same transformation as applied to the point estimate.

### Limitations

This simulation study has four possible limitations. First, our continuous norming models included only one predictor (i.e., age). The interpretation of the normed scores crucially depends on the used predictor(s), because the predictor(s) fully determine the reference populations. For intelligence and developmental tests, the reference population typically is the general population of the same age, implying that in continuous norming age will be the only predictor. However, in different types of tests, one may have additional predictor(s). For example, in clinical tests, one may have a healthy population of the same age, sex, and education level as the reference population. Continuous norming of such a test would thus require the predictors’ age, sex, and educational level. The used continuous norming models can easily be extended to include more predictors. Categorical predictors can be included using dummy coding (e.g., Cohen et al., 2003). We believe that using more predictors would have complicated our simulation study unnecessarily, as we expect similar results for models with more predictors, including categorical ones. Note that adding predictors may complicate the model, rendering a larger sample necessary to achieve the same precision.

Second, we sampled the observations randomly from each population model. Therefore, the expected distribution of age within a sample is uniform. We refrained from varying the distribution of the predictor values, because their effects on precision were beyond our interest, and their effects heavily depends on the specific population model. That is, the effects of such an unbalanced design on the quality of the estimated model depends on the nature of the longitudinal model, which describes the relationships between age and the distributional parameters of the GAMLSS. Generally, the more complicated the relationship is, the larger the effect of unbalancedness will be. For example, linear relationships could be approximated well based on only observations near the boundaries of the age range, while higher order polynomials would also need support by observations within the age range.

Third, we used a limited number of population models and estimation models. We could have generated assumption violations in different ways (e.g., violation of the normality assumption with a bimodal distribution), and used other estimation models (e.g., for ordered categorical data). However, we carefully manipulated the characteristics of the population models in terms of their longitudinal and transversal nature, and matched with a series of estimation models that could be presumed to be used in practice. For example, using an estimation model for ordered categorical data will probably not used for continuous empirical data, hence would be rather far-fetched to examine.

Fourth, we only explored estimation through GAMLSS to deal with violated assumptions of the standard regression model. Alternatives are semiparametric continuous norming (Lenhard et al., 2018) and robust regression (Wilcox, 2012). The semiparametric approach performed similar to the use of specific GAMLSS distributions, except for extreme (i.e., easy and difficult) tests for the population of interest, where the semiparametric approach outperformed (Lenhard et al., 2019). Considering robust regression, Oosterhuis (2017) used the distribution-free Harrell–Davis (Harrell & Davis, 1982) quantile estimator to estimate percentiles without assuming normality of the conditional score distribution. This required the unrealistic assumption that the shape of the score distribution was consistent across the predictor range. Such alternative approaches could have a different bias-variance trade-off than the models studied in this article.

### Relationships Between Norming and Measurement Models

Normed scores are based on raw test scores. To arrive at the transformation rules, one only needs the distribution(s) of the raw test scores in the reference population(s). This implies that no assumptions are made on what is actually measured by the test. This leaves aside that normed scores only make sense if the raw test scores provide a reasonable quantification of the construct of interest. This issue can be approached using psychometric theory, including classical test theory and latent variable theory (e.g., Raykov & Marcoulides, 2011), complemented by appropriate validation studies.

If a test score is composed of individual item scores, a latent variable model (e.g., common factor model or item response theory-based model) is of use to assess the quality of the items and the resulting test. Furthermore, this would offer the possibility to examine possible violations of measurement invariance (e.g., Vandenberg & Lance, 2000). For normed scores, it is only necessary to consider violators of measurement invariance that vary among individuals within the reference population(s) of the test. Thus, it would make sense to assess measurement invariance for sex for an intelligence test, but not for a neuropsychological test with sex specific norms.

## Supplemental Material

Supplementary_Tables_and_Figure – Supplemental material for Bias-Variance Trade-Off in Continuous Test Norming Click here for additional data file. Supplemental material, Supplementary_Tables_and_Figure for Bias-Variance Trade-Off in Continuous Test Norming by Lieke Voncken, Casper J. Albers and Marieke E. Timmerman in Assessment
