# Supplementary material for: Bias-Variance Trade-Off in Continuous Test Norming
Source: Assessment. 2020 Jul 13;28(8):1932–48. doi: 10.1177/1073191120939155 (PMC8543664; doi:10.1177/1073191120939155)
Supplement: Supplementary_Tables_and_Figure – Supplemental material for Bias-Variance Trade-Off in Continuous Test Norming [file Supplementary_Tables_and_Figure.pdf]

Table S1: Population models in the simulation study

Characteristic of population model		Model
Longitudinal	Linear (Li)	$\mu = 34.0 + 344.9\, f_1(\text{age})$
	Non-linear (NL)	$\mu = 34.0 + 342.9\, f_1(\text{age}) - 114.2\, f_2(\text{age}) + 14.6\, f_3(\text{age}) + 24.9\, f_4(\text{age})$
Transversal	Homoscedastic (Ho)	$\ln(\sigma) = 2.0$
	Heteroscedastic (He)	$\ln(\sigma) = 1.8 + 12.3\, f_1(\text{age}) - 6.9\, f_2(\text{age})$
	Normal (No)	$\mathcal{N}(\mu, \sigma)$
	Non-normal (NN)	$SST(\mu, \sigma, \nu, \tau)$
		$\ln(\nu) = -9.6\, f_1(\text{age}) + 4.1\, f_2(\text{age}) + 6.2\, f_3(\text{age}) - 3.9\, f_4(\text{age})$
		$\ln(\tau - 2) = 3.9 + 83.4\, f_1(\text{age})$

Note. $\mathcal{N}(\mu, \sigma)$ refers to the normal distribution with distributional parameters μ for the mean and σ for the standard deviation. $SST(\mu, \sigma, \nu, \tau)$ refers to the skew Student t distribution with distributional parameters μ for the mean, σ for the standard deviation, and ν and τ for the skewness and kurtosis. $f_d(\text{age})$ refers to an orthogonal polynomial of age of degree d. The model parameters are rounded to one decimal place.

Table S2: Mean RMSE (SE) for the population models and estimation models, for $N = 500$, across all age values and test scores.

Estimation model	Strict			True		Flex			BCPE
	Long+Trans	Long	Trans	Linear/ Splines	Poly	Trans	Long	Long+Trans	Long+Trans
NL-HeNN	0.503 (0.001)	0.476 (<0.001)	0.392 (0.001)	0.365 (0.002)	0.221 (<0.001)	-	-	-	0.304 (0.001)
Li-HeNN	-	-	0.382 (0.001)	0.213 (<0.001)	-	-	0.214 (<0.001)	-	0.335 (0.001)
NL-HoNN	0.388 (0.001)	0.409 (0.001)	0.254 (0.001)	0.194 (<0.001)	-	0.306 (0.002)	-	-	0.965 (0.004)
Li-HoNN	-	-	0.240 (0.001)	0.175 (<0.001)	-	0.401 (0.002)	0.175 (<0.001)	0.349 (0.002)	0.743 (0.004)
NL-HeNo	0.511 (0.001)	0.440 (0.001)	0.386 (0.001)	0.251 (0.001)	-	1.431 (0.004)	-	-	0.265 (0.001)
Li-HeNo	-	-	0.371 (0.001)	0.233 (0.001)	-	1.333 (0.004)	0.233 (0.001)	1.382 (0.004)	0.270 (<0.001)
NL-HoNo	-	0.362 (0.001)	-	0.116 (<0.001)	-	1.748 (0.004)	-	-	0.188 (<0.001)
Li-HoNo	-	-	-	0.082 (<0.001)	-	1.927 (0.004)	0.082 (<0.001)	2.029 (0.003)	0.153 (<0.001)

Note. The population models are either nonlinear (NL) or linear (Li), either homoscedastic (Ho) or heteroscedastic (He), and either normal (No) or non-normal (NN). The estimation models are too strict or too flexible (flex) related to the trans(versal) and/or long(itudinal) model. In addition, a flexible Box-Cox Power Exponential (BCPE) model is estimated.

Table S3: Mean RMSE (SE) for the population models and estimation models, for N = 1,000, across all age values and test scores.

Estimation model	Strict			True		Flex			BCPE
	Long+Trans	Long	Trans	Linear/ Splines	Poly	Trans	Long	Long+Trans	Long+Trans
NL-HeNN	0.498 (0.001)	0.419 (0.001)	0.378 (0.001)	0.164 (<0.001)	0.135 (<0.001)	-	-	-	0.290 (0.002)
Li-HeNN	-	-	0.372 (0.001)	0.145 (<0.001)	-	-	0.146 (<0.001)	-	0.281 (0.001)
NL-HoNN	0.381 (0.001)	0.397 (0.001)	0.236 (0.001)	0.139 (<0.001)	-	0.146 (<0.001)	-	-	1.188 (0.004)
Li-HoNN	-	-	0.226 (0.001)	0.120 (<0.001)	-	0.128 (<0.001)	0.121 (<0.001)	0.128 (<0.001)	1.340 (0.004)
NL-HeNo	0.505 (0.001)	0.437 (0.001)	0.372 (0.001)	0.196 (<0.001)	-	1.223 (0.004)	-	-	0.224 (<0.001)
Li-HeNo	-	-	0.361 (0.001)	0.179 (<0.001)	-	1.439 (0.004)	0.179 (<0.001)	1.670 (0.004)	0.229 (0.001)
NL-HoNo	-	0.355 (0.001)	-	0.085 (<0.001)	-	1.713 (0.004)	-	-	0.126 (<0.001)
Li-HoNo	-	-	-	0.058 (<0.001)	-	1.535 (0.004)	0.058 (<0.001)	1.671 (0.004)	0.102 (<0.001)

Note. The population models are either nonlinear (NL) or linear (Li), either homoscedastic (Ho) or heteroscedastic (He), and either normal (No) or non-normal (NN). The estimation models are too strict or too flexible (flex) related to the trans(versal) and/or long(itudinal) model. In addition, a flexible Box-Cox Power Exponential (BCPE) model is estimated.

Table S4: Mean RMSE (SE) for the population models and estimation models, for $N = 2{,}000$, across all age values and test scores.

Estimation model	Strict			True		Flex			BCPE
	Long+Trans	Long	Trans	Linear/ Splines	Poly	Trans	Long	Long+Trans	Long+Trans
NL-HeNN	0.496 (0.001)	0.401 (0.001)	0.372 (0.001)	0.124 (<0.001)	0.091 (<0.001)	-	-	-	0.305 (0.002)
Li-HeNN	-	-	0.366 (0.001)	0.094 (<0.001)	-	-	0.095 (<0.001)	-	0.327 (0.002)
NL-HoNN	0.372 (0.001)	0.402 (0.001)	0.227 (0.001)	0.110 (<0.001)	-	0.114 (<0.001)	-	-	1.567 (0.004)
Li-HoNN	-	-	0.218 (0.001)	0.088 (<0.001)	-	0.084 (<0.001)	0.088 (<0.001)	0.081 (<0.001)	1.269 (0.004)
NL-HeNo	0.511 (0.001)	0.418 (0.001)	0.367 (0.001)	0.116 (<0.001)	-	0.352 (0.002)	-	-	0.169 (<0.001)
Li-HeNo	-	-	0.356 (0.001)	0.080 (<0.001)	-	0.923 (0.004)	0.080 (<0.001)	1.056 (0.004)	0.140 (<0.001)
NL-HoNo	-	0.354 (0.001)	-	0.085 (<0.001)	-	0.862 (0.004)	-	-	0.094 (<0.001)
Li-HoNo	-	-	-	0.041 (<0.001)	-	1.221 (0.004)	0.041 (<0.001)	1.138 (0.004)	0.068 (<0.001)

Note. The population models are either nonlinear (NL) or linear (Li), either homoscedastic (Ho) or heteroscedastic (He), and either normal (No) or non-normal (NN). The estimation models are too strict or too flexible (flex) related to the trans(versal) and/or long(itudinal) model. In addition, a flexible Box-Cox Power Exponential (BCPE) model is estimated.

Table S5: Mean absolute bias (SE) for the population models and estimation models, for $N = 500$, across all age values and test scores.

Estimation model	Strict			True		Flex			BCPE
	Long+Trans	Long	Trans	Linear/ Splines	Poly	Trans	Long	Long+Trans	Long+Trans
NL-HeNN	0.492 (0.001)	0.373 (0.001)	0.361 (0.001)	0.203 (0.001)	0.044 (<0.001)	-	-	-	0.121 (<0.001)
Li-HeNN	-	-	0.361 (0.001)	0.090 (<0.001)	-	-	0.090 (<0.001)	-	0.151 (0.001)
NL-HoNN	0.372 (0.001)	0.356 (0.001)	0.214 (0.001)	0.093 (<0.001)	-	0.167 (0.002)	-	-	0.670 (0.003)
Li-HoNN	-	-	0.208 (0.001)	0.082 (<0.001)	-	0.243 (0.002)	0.082 (<0.001)	0.210 (0.002)	0.536 (0.003)
NL-HeNo	0.500 (0.001)	0.416 (0.001)	0.359 (0.001)	0.203 (0.001)	-	1.086 (0.003)	-	-	0.178 (<0.001)
Li-HeNo	-	-	0.352 (0.001)	0.191 (0.001)	-	1.019 (0.003)	0.191 (0.001)	1.071 (0.003)	0.191 (0.001)
NL-HoNo	-	0.347 (0.001)	-	0.055 (<0.001)	-	1.289 (0.003)	-	-	0.076 (<0.001)
Li-HoNo	-	-	-	0.005 (<0.001)	-	1.456 (0.003)	0.005 (<0.001)	1.490 (0.003)	0.033 (<0.001)

Note. The population models are either nonlinear (NL) or linear (Li), either homoscedastic (Ho) or heteroscedastic (He), and either normal (No) or non-normal (NN). The estimation models are too strict or too flexible (flex) related to the trans(versal) and/or long(itudinal) model. In addition, a flexible Box-Cox Power Exponential (BCPE) model is estimated.

Table S6: Mean absolute bias (SE) for the population models and estimation models, for N = 1,000, across all age values and test scores.

Estimation model	Strict			True		Flex			BCPE
	Long+Trans	Long	Trans	Linear/ Splines	Poly	Trans	Long	Long+Trans	Long+Trans
NL-HeNN	0.491 (0.001)	0.366 (0.001)	0.359 (0.001)	0.075 (<0.001)	0.019 (<0.001)	-	-	-	0.155 (0.001)
Li-HeNN	-	-	0.361 (0.001)	0.059 (<0.001)	-	-	0.059 (<0.001)	-	0.144 (0.001)
NL-HoNN	0.372 (0.001)	0.363 (0.001)	0.211 (0.001)	0.060 (<0.001)	-	0.061 (<0.001)	-	-	0.926 (0.004)
Li-HoNN	-	-	0.208 (0.001)	0.047 (<0.001)	-	0.048 (<0.001)	0.047 (<0.001)	0.048 (<0.001)	1.004 (0.003)
NL-HeNo	0.499 (0.001)	0.410 (0.001)	0.355 (0.001)	0.129 (<0.001)	-	1.019 (0.004)	-	-	0.173 (<0.001)
Li-HeNo	-	-	0.350 (0.001)	0.118 (<0.001)	-	1.124 (0.003)	0.118 (<0.001)	1.273 (0.003)	0.181 (0.001)
NL-HoNo	-	0.347 (0.001)	-	0.040 (<0.001)	-	1.333 (0.004)	-	-	0.051 (<0.001)
Li-HoNo	-	-	-	0.004 (<0.001)	-	1.162 (0.003)	0.004 (<0.001)	1.265 (0.003)	0.016 (<0.001)

Note. The population models are either nonlinear (NL) or linear (Li), either homoscedastic (Ho) or heteroscedastic (He), and either normal (No) or non-normal (NN). The estimation models are too strict or too flexible (flex) related to the trans(versal) and/or long(itudinal) model. In addition, a flexible Box-Cox Power Exponential (BCPE) model is estimated.

Table S7: Mean absolute bias (SE) for the population models and estimation models, for $N = 2{,}000$, across all age values and test scores.

Estimation model	Strict			True		Flex			BCPE
	Long+Trans	Long	Trans	Linear/ Splines	Poly	Trans	Long	Long+Trans	Long+Trans
NL-HeNN	0.493 (0.001)	0.363 (0.001)	0.360 (0.001)	0.069 (<0.001)	0.010 (<0.001)	-	-	-	0.172 (0.002)
Li-HeNN	-	-	0.360 (0.001)	0.036 (<0.001)	-	-	0.036 (<0.001)	-	0.204 (0.002)
NL-HoNN	0.367 (0.001)	0.371 (0.001)	0.213 (0.001)	0.062 (<0.001)	-	0.062 (<0.001)	-	-	1.177 (0.003)
Li-HoNN	-	-	0.208 (0.001)	0.023 (<0.001)	-	0.024 (<0.001)	0.023 (<0.001)	0.024 (<0.001)	0.949 (0.003)
NL-HeNo	0.508 (0.001)	0.387 (0.001)	0.357 (0.001)	0.070 (<0.001)	-	0.228 (0.002)	-	-	0.107 (<0.001)
Li-HeNo	-	-	0.350 (0.001)	0.030 (<0.001)	-	0.668 (0.003)	0.030 (<0.001)	0.802 (0.003)	0.067 (<0.001)
NL-HoNo	-	0.349 (0.001)	-	0.059 (<0.001)	-	0.710 (0.003)	-	-	0.043 (<0.001)
Li-HoNo	-	-	-	0.001 (<0.001)	-	0.915 (0.003)	0.001 (<0.001)	0.831 (0.003)	0.007 (<0.001)

Note. The population models are either nonlinear (NL) or linear (Li), either homoscedastic (Ho) or heteroscedastic (He), and either normal (No) or non-normal (NN). The estimation models are too strict or too flexible (flex) related to the trans(versal) and/or long(itudinal) model. In addition, a flexible Box-Cox Power Exponential (BCPE) model is estimated.

Table S8: Mean variance (SE) for the population models and estimation models, for $N = 500$, across all age values and test scores.

Estimation model	Strict			True		Flex			BCPE
	Long+Trans	Long	Trans	Linear/ Splines	Poly	Trans	Long	Long+Trans	Long+Trans
NL-HeNN	0.006 (<0.001)	0.086 (<0.001)	0.011 (<0.001)	0.297 (0.004)	0.068 (<0.001)	-	-	-	0.112 (0.001)
Li-HeNN	-	-	0.008 (<0.001)	0.051 (<0.001)	-	-	0.052 (<0.001)	-	0.198 (0.003)
NL-HoNN	0.007 (<0.001)	0.036 (<0.001)	0.013 (<0.001)	0.038 (<0.001)	-	0.169 (0.002)	-	-	0.991 (0.008)
Li-HoNN	-	-	0.009 (<0.001)	0.033 (<0.001)	-	0.390 (0.005)	0.033 (<0.001)	0.308 (0.005)	0.693 (0.007)
NL-HeNo	0.006 (<0.001)	0.012 (<0.001)	0.010 (<0.001)	0.020 (<0.001)	-	1.293 (0.007)	-	-	0.042 (<0.001)
Li-HeNo	-	-	0.007 (<0.001)	0.018 (<0.001)	-	1.270 (0.007)	0.018 (<0.001)	1.222 (0.007)	0.042 (<0.001)
NL-HoNo	-	0.006 (<0.001)	-	0.010 (<0.001)	-	1.527 (0.006)	-	-	0.039 (<0.001)
Li-HoNo	-	-	-	0.007 (<0.001)	-	1.809 (0.007)	0.007 (<0.001)	2.043 (0.007)	0.033 (<0.001)

Note. The population models are either nonlinear (NL) or linear (Li), either homoscedastic (Ho) or heteroscedastic (He), and either normal (No) or non-normal (NN). The estimation models are too strict or too flexible (flex) related to the trans(versal) and/or long(itudinal) model. In addition, a flexible Box-Cox Power Exponential (BCPE) model is estimated.

Table S9: Mean variance (SE) for the population models and estimation models, for $N = 1{,}000$, across all age values and test scores.

Estimation model	Strict			True		Flex			BCPE
	Long+Trans	Long	Trans	Linear/ Splines	Poly	Trans	Long	Long+Trans	Long+Trans
NL-HeNN	0.003 (<0.001)	0.033 (<0.001)	0.006 (<0.001)	0.027 (<0.001)	0.024 (<0.001)	-	-	-	0.150 (0.003)
Li-HeNN	-	-	0.004 (<0.001)	0.024 (<0.001)	-	-	0.025 (<0.001)	-	0.148 (0.003)
NL-HoNN	0.004 (<0.001)	0.020 (<0.001)	0.007 (<0.001)	0.020 (<0.001)	-	0.023 (<0.001)	-	-	1.030 (0.007)
Li-HoNN	-	-	0.005 (<0.001)	0.017 (<0.001)	-	0.020 (<0.001)	0.017 (<0.001)	0.020 (<0.001)	1.378 (0.008)
NL-HeNo	0.003 (<0.001)	0.015 (<0.001)	0.006 (<0.001)	0.026 (<0.001)	-	0.836 (0.006)	-	-	0.020 (<0.001)
Li-HeNo	-	-	0.004 (<0.001)	0.024 (<0.001)	-	1.260 (0.007)	0.024 (<0.001)	1.526 (0.007)	0.021 (<0.001)
NL-HoNo	-	0.003 (<0.001)	-	0.006 (<0.001)	-	1.403 (0.007)	-	-	0.016 (<0.001)
Li-HoNo	-	-	-	0.004 (<0.001)	-	1.364 (0.007)	0.004 (<0.001)	1.492 (0.007)	0.014 (<0.001)

Note. The population models are either nonlinear (NL) or linear (Li), either homoscedastic (Ho) or heteroscedastic (He), and either normal (No) or non-normal (NN). The estimation models are too strict or too flexible (flex) related to the trans(versal) and/or long(itudinal) model. In addition, a flexible Box-Cox Power Exponential (BCPE) model is estimated.

Table S10: Mean variance (SE) for the population models and estimation models, for $N = 2{,}000$, across all age values and test scores.

Estimation model	Strict			True		Flex			BCPE
	Long+Trans	Long	Trans	Linear/ Splines	Poly	Trans	Long	Long+Trans	Long+Trans
NL-HeNN	0.002 (<0.001)	0.022 (<0.001)	0.003 (<0.001)	0.012 (<0.001)	0.011 (<0.001)	-	-	-	0.130 (0.002)
Li-HeNN	-	-	0.002 (<0.001)	0.010 (<0.001)	-	-	0.010 (<0.001)	-	0.226 (0.004)
NL-HoNN	0.002 (<0.001)	0.019 (<0.001)	0.004 (<0.001)	0.009 (<0.001)	-	0.010 (<0.001)	-	-	1.601 (0.008)
Li-HoNN	-	-	0.002 (<0.001)	0.009 (<0.001)	-	0.009 (<0.001)	0.009 (<0.001)	0.008 (<0.001)	1.315 (0.008)
NL-HeNo	0.002 (<0.001)	0.019 (<0.001)	0.003 (<0.001)	0.009 (<0.001)	-	0.282 (0.004)	-	-	0.022 (<0.001)
Li-HeNo	-	-	0.002 (<0.001)	0.007 (<0.001)	-	0.843 (0.006)	0.007 (<0.001)	0.927 (0.006)	0.022 (<0.001)
NL-HoNo	-	0.002 (<0.001)	-	0.003 (<0.001)	-	0.649 (0.006)	-	-	0.008 (<0.001)
Li-HoNo	-	-	-	0.002 (<0.001)	-	1.015 (0.006)	0.002 (<0.001)	0.999 (0.006)	0.006 (<0.001)

Note. The population models are either nonlinear (NL) or linear (Li), either homoscedastic (Ho) or heteroscedastic (He), and either normal (No) or non-normal (NN). The estimation models are too strict or too flexible (flex) related to the trans(versal) and/or long(itudinal) model. In addition, a flexible Box-Cox Power Exponential (BCPE) model is estimated.

Table S11: Nonconvergence for the population models and estimation models, and for $N = 500$

Estimation model	Strict			True		Flex			BCPE
	Long+Trans	Long	Trans	Linear/ Splines	Poly	Trans	Long	Long+Trans	Long+Trans
NL-HeNN	0/0	0/0	0/0	0/0	0/0	-	-	-	0/17
Li-HeNN	-	-	1/0	1/9	-	-	1/9	-	0/7
NL-HoNN	0/0	0/11	0/0	0/57	-	0/45	-	-	0/9
Li-HoNN	-	-	1/0	1/10	-	1/60	1/9	1/7	0/2
NL-HeNo	0/0	0.0	0/0	0/0	-	0/20	-	-	0/17
Li-HeNo	-	-	1/0	1/0	-	1/0	1/0	1/6	0/2
NL-HoNo	-	0.0	-	0/0	-	0/29	-	-	0/1
Li-HoNo	-	-	-	1/0	-	1/0	1/0	1/5	0/1

Note. The first value represents the number of replications in which the model could not be estimated; the second value represents the number of replications in which the maximum number of iterations (i.e., 2,000) was achieved in the model estimation. The total number of replications per condition was 1,000.

Table S12: Nonconvergence for the population models and estimation models, and for N = 1,000

Estimation model	Strict			True		Flex			BCPE
	Long+Trans	Long	Trans	Linear/ Splines	Poly	Trans	Long	Long+Trans	Long+Trans
NL-HeNN	1/0	1/3	1/0	1/0	0/0	-	-	-	0/33
Li-HeNN	-	-	1/0	1/15	-	-	1/10	-	0/11
NL-HoNN	1/0	1/14	1/0	1/36	-	1/39	-	-	0/14
Li-HoNN	-	-	1/0	1/18	-	1/36	1/17	1/17	0/4
NL-HeNo	1/0	1/0	1/0	1/0	-	1/35	-	-	0/10
Li-HeNo	-	-	1/0	1/0	-	1/0	1/0	1/26	0/2
NL-HoNo	-	1/0	-	1/0	-	1/34	-	-	0/0
Li-HoNo	-	-	-	1/0	-	1/0	1/0	1/1	0/0

Note. The first value represents the number of replications in which the model could not be estimated; the second value represents the number of replications in which the maximum number of iterations (i.e., 2,000) was achieved in the model estimation. The total number of replications per condition was 1,000.

Table S13: Nonconvergence for the population models and estimation models, and for $N = 2{,}000$

Estimation model	Strict			True		Flex			BCPE
	Long+Trans	Long	Trans	Linear/ Splines	Poly	Trans	Long	Long+Trans	Long+Trans
NL-HeNN	3/0	3/3	3/0	3/0	0/0	-	-	-	0/25
Li-HeNN	-	-	4/0	4/1	-	-	4/6	-	0/13
NL-HoNN	3/0	3/6	3/0	3/17	-	3/20	-	-	0/28
Li-HoNN	-	-	4/0	4/17	-	4/17	4/13	4/17	0/7
NL-HeNo	3/0	3/0	3/0	3/0	-	3/45	-	-	0/2
Li-HeNo	-	-	4/0	4/0	-	4/0	4/3	4/43	0/0
NL-HoNo	-	3/0	-	3/0	-	3/28	-	-	0/0
Li-HoNo	-	-	-	4/0	-	4/3	4/0	4/1	0/0

Note. The first value represents the number of replications in which the model could not be estimated; the second value represents the number of replications in which the maximum number of iterations (i.e., 2,000) was achieved in the model estimation. The total number of replications per condition was 1,000.

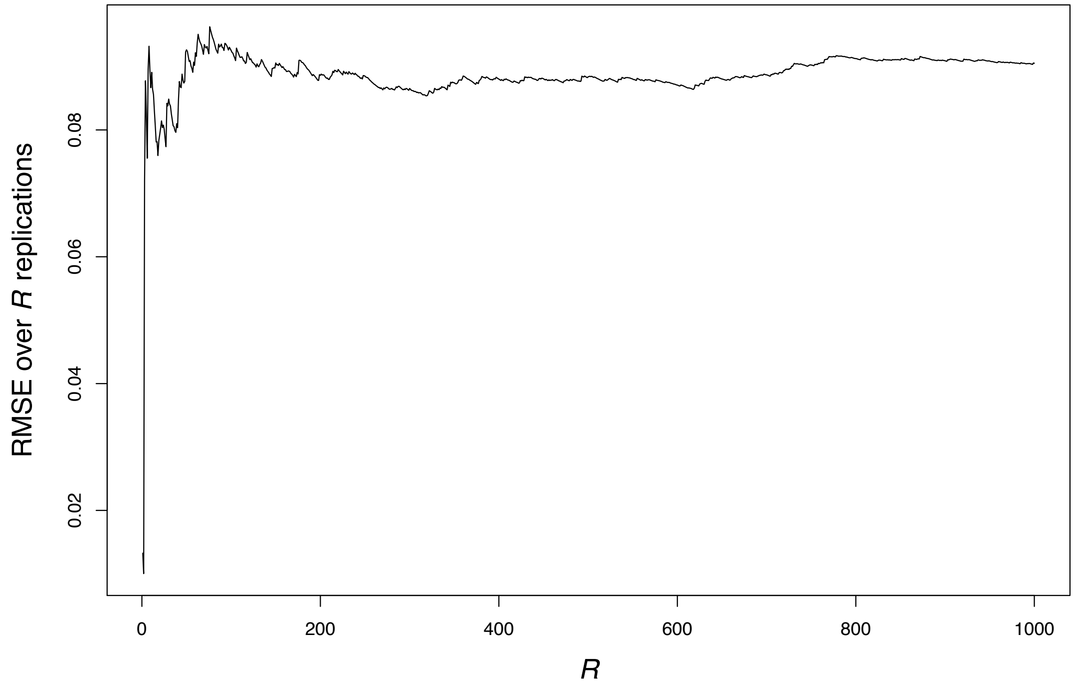

Figure S1. RMSE over R replications, for the condition with a linear, homoscedastic, and normal population model (Li-HoNo), the true estimation model, $N = 500$, age = 5, and z score = 0.
